# Psychometric properties of the Automatic Thoughts Questionnaire-8 in two Spanish nonclinical samples

**DOI:** 10.7717/peerj.9747

**Published:** 2020-09-16

**Authors:** Francisco J. Ruiz, Miguel A. Segura-Vargas, Paula Odriozola-González, Juan C. Suárez-Falcón

**Affiliations:** 1 Fundación Universitaria Konrad Lorenz, Bogotá, Colombia; 2 Universidad de Valladolid, Valladolid, Spain; 3 Universidad Nacional de Educación a Distancia (UNED), Madrid, Spain

**Keywords:** Automatic Thoughts Questionnaire-8, Confirmatory factor analysis, Depression, Negative automatic thoughts

## Abstract

**Background:**

The ATQ is a widely used instrument consisting of 30 items that assess the frequency of negative automatic thoughts. However, the extensive length of the ATQ could compromise its measurement efficiency in survey research. Consequently, an 8-item shortened version of the ATQ has been developed. This study aims to analyze the validity of the ATQ-8 in two Spanish samples.

**Method:**

The ATQ-8 was administered to a total sample of 1,148 participants (302 undergraduates and 846 general online population). To analyze convergent construct validity, the questionnaire package also included the Dysfunctional Attitude Scale-Revised (DAS-R), Depression Anxiety and Stress Scale-21 (DASS-21), Acceptance Action Questionnaire-II (AAQ-II), Cognitive Fusion Questionnaire (CFQ), Generalized Pliance Questionnaire (GPQ), and Satisfaction with Life Scale (SWLS). To analyze internal consistency, we computed Cronbach’s alpha and McDonald’s omega. A confirmatory factor analysis was conducted to test the one-factor structure of the ATQ-8. In so doing, a robust diagonally weighted least square estimation method (Robust DWLS) was adopted using polychoric correlations. Afterward, we analyzed measurement invariance across samples, gender, groupage, and education level. Lastly, we evaluated convergent construct validity by computing Pearson correlations between the ATQ-8 and the remaining instruments.

**Results:**

The internal consistency across samples was adequate (alpha and omega = .89). The one-factor model demonstrated a good fit to the data (RMSEA = 0.10, 90% CI [0.089, 0.112], CFI = 0.98, NNFI = 0.97, and SRMR = 0.048). The ATQ-8 showed scalar metric invariance across samples, gender, groupage, and education level. The ATQ-8 scores were significantly associated with emotional symptoms (DASS-21), satisfaction with life (SWLS), dysfunctional schemas (DAS-R), cognitive fusion (CFQ), experiential avoidance (AAQ-II), and generalized pliance (GPQ). In conclusion, the Spanish version of the ATQ-8 demonstrated adequate psychometric properties in Spanish samples.

## Introduction

Unipolar depression is characterized by sadness, irritability, or anhedonia, as well as a loss of appetite, difficulty to sleep, fatigue, slowing of speech and action, and suicidal thoughts, among others (American Psychiatric Association, 2013). The cognitive model proposed by Beck et al. (1979) states that the cognitive triad, integrated by a pattern of negative thinking about the world, the future, and the self, is one of the pillars of depression. Within this cognitive pattern, negative automatic thoughts play a crucial role and are defined as negative self-statements (Beck et al., 1979).

The Automatic Thoughts Questionnaire is one of the most extensively used instruments to measure negative automatic thoughts (ATQ; Hollon & Kendall, 1980). The ATQ is an instrument consisting of 30 items with a 5-point Likert scale that assesses the frequency of negative automatic thoughts experienced during the past week. Hollon & Kendall (1980) asked 312 undergraduates to recall dysphoric experiences and to report associated cognitions. Afterward, the authors chose 100 representative cognitions and administered them to a second sample. Through a cross-validation analysis, the authors retained 30 of the 100 original items. These items significantly discriminated between clinical and nonclinical samples (Hollon & Kendall, 1980).

Several studies have confirmed the temporal consistency, convergent and discriminant validity, and excellent internal consistency of the ATQ (e.g., Chioqueta & Stiles, 2004; Hollon & Kendall, 1980; Hollon, Kendall & Lumry, 1986; Kazdin, 1990). The results of exploratory factor analyses across different studies yielded factor structures with more than one factor (e.g., Bryant & Baxter, 1997; Cano-García & Rodríguez-Franco, 2002; Chioqueta & Stiles, 2006; Deardorff, Hopkins & Finch Jr, 1984; Ghassemzadeh et al., 2005; Joseph, 1994; Kazdin, 1990; Oei & Mukhtar, 2008; Sahin & Sahin, 1992; see reviews in Netemeyer et al., 2002; Zettle et al., 2013). Most studies have obtained different factor solutions from the four factors shown by Hollon & Kendall (1980). Netemeyer et al. (2002) mentioned that all studies found that a large proportion of the variance was accounted for the first factor. Consequently, the results suggest that one factor could underlie the 30 items of the ATQ. Moreover, most studies have only used the overall score of the ATQ, which treats the scale as if it were only represented by one factor.

The extensive length of the ATQ could compromise its measurement efficiency in survey research. Accordingly, Netemeyer et al. (2002) gathered two samples (*N* = 434 and *N* = 419) to derive the 15- and 8-item reduced versions of the ATQ. Both versions of the questionnaire had a single factor, with alphas of .96 and .92, respectively. Two additional cross-validation samples (*N* = 163 and *N* = 91) also showed support for the 15-and 8-item reduced versions, which suggests that the shortened versions of the ATQ are suitable alternatives to measure automatic cognitions associated with depression (Netemeyer et al., 2002).

Following the study by Netemeyer et al. (2002), Ruiz, Suárez-Falcón & Riaño Hernández (2017) analyzed the psychometric properties of the Spanish version of the ATQ-8 in a Colombian sample of 1,587 participants, including general population, a clinical sample, and undergraduates. The analysis displayed good internal consistency across samples (alpha of. 89), and the one-factor model obtained an adequate fit to the data (RMSEA = 0.083, 90% CI [0.074, 0.092]; CFI = .96; NNFI = .95). Additional factor analyses confirmed measurement invariance across gender and samples (i.e., clinical and nonclinical samples). Furthermore, the mean scores of the clinical sample were significantly higher than the scores of their nonclinical counterpart.

The results presented in Netemeyer et al. (2002) and Ruiz et al. (2017b) indicate that the ATQ-8 might be an excellent alternative to the original ATQ scale. However, the factor structure and psychometric properties of the ATQ-8 have been analyzed only in two countries. Accordingly, the current study aims to analyze the validity of the ATQ-8 in Spaniard samples. This study is relevant because the original ATQ was only preliminarily validated in Spain by Cano-García & Rodríguez-Franco (2002) in a sample of 205 individuals suffering from chronic pain. Thus, there is scarce empirical evidence of the psychometric properties of the ATQ in nonclinical samples in Spain.

This study analyzes the factor structure and psychometric properties of the ATQ-8 in two nonclinical Spanish samples. The first sample consisted of 302 undergraduates and the second one of 846 individuals from the general population.

## Materials & Methods

The procedures followed in the research reported in the manuscript were approved by the Bioethics Committee of Fundación Universitaria Konrad Lorenz (2016-021B). Written informed consent was obtained from all participants in this study.

### Participants

*Sample 1*. This sample consisted of 302 undergraduates (age range 18–61, *M* = 26.18, *SD* = 9.75, 64.6% of females) from a Spanish university. Of the overall sample, 4.3% of the participants were currently in treatment, 19.4% had received psychological or psychiatric treatment, and 3.7% were taking psychotropic medication.

*Sample 2*. This sample consisted of 846 participants from general population, who completed the instruments online (age range 18-72, *M* = 35.14, *SD* = 11.39, 75.7% of females). Of the overall sample, 3.4% of participants had completed primary studies, 31% secondary studies, and 55.6% were university graduates. Also, 12.8% of participants were currently in treatment, 44.6% had received psychological or psychiatric treatment, and 12.9% were taking psychotropic medication.

### Instruments

*Automatic Thoughts Questionnaire-8* (ATQ-8; Netemeyer et al., 2002; Spanish version by Cano-García & Rodríguez-Franco, 2002). The ATQ-8 is the reduced version of the ATQ. Through a Likert-type scale (5 = *all the time*; 1 = *not at all*), it measures the frequency of negative thoughts during the past week. Examples of items are “I’m so disappointed in myself”, “I feel so helpless”, “My future is bleak”, and “I can’t finish anything”.

*Dysfunctional Attitude Scale-Revised* (DAS-R; De Graaf, Roelofs & Huibers, 2009; Spanish version by Ruiz et al., 2015). The DAS is a traditional instrument that measures dysfunctional schemas. Its revised version (i.e., DAS-R) has 17 items, which are responded on a 7-point Likert-type scale (7 = *fully agree*; 1 = *fully disagree*), organized into two factors: Perfectionism/Performance evaluation and Dependency. Examples of the items are: “If a person asks for help, it is a sign of weakness”, “My happiness depends more on other people than it does on me”, “If I fail at my work, then I am a failure as a person”, and “If others dislike you, you cannot be happy”. The DAS-R has shown a factor structure with two correlated factors and a second-order factor and has also demonstrated adequate psychometric properties in Spanish and Colombian samples (Ruiz et al., 2016; Ruiz et al., 2015). In this study, the DAS-R presented a Cronbach’s alpha of .88 in Sample 1. According to the cognitive model of depression, medium to strong correlations were expected between the DAS-R and the ATQ-8.

*Depression, Anxiety, and Stress Scales-21* (DASS-21; Lovibond & Lovibond, 1995; Spanish version by Daza et al., 2002). The DASS-21 measures negative emotional states experienced during the last week through 21 items on a 4-point Likert-type scale (3 = *applied to me very much or most of the time*; 0 = *did not apply to me at all*). Examples of the items are: “I couldn’t experience positive feeling”, “I felt close to panic”, and “I found it difficult to relax”. The DASS-21 has shown a hierarchical factor structure consisting of three first-order factors (Depression, Anxiety, and Stress) and a second-order factor. The latter can be considered as an overall indicator of emotional symptoms (Ruiz et al., 2017a). The DASS-21 has also presented good convergent and discriminant validity and internal consistency. Alpha values in this study for the DASS-Total were .92 and .95 for Sample 1 and 2, respectively. The DASS-21 was administered because, in previous studies, emotional symptoms and not only depression have been strongly associated with the frequency of negative thoughts. Consequently, strong correlations were expected between the DASS-21 subscales and the ATQ-8.

*Satisfaction with Life Scale* (SWLS; Diener et al., 1985; Spanish version by Atienza et al., 2000). The SWLS measures self-perceived well-being through 5 items, graded with a 7-point Likert-type scale (7 = *strongly agree*; 1 = *strongly disagree*). Examples of items are “If I could live my life over, I would change almost nothing”, “In most ways, my life is close to my ideal”, and “The conditions in my life are excellent”. The SWLS has demonstrated adequate convergent validity and psychometric properties. Alpha values in the study were .84 and .89 for Samples 1 and 2, respectively. Previous research has demonstrated that the frequency of negative thoughts is negatively associated with life satisfaction (Ruiz et al., 2017b). Medium to strong negative correlations were expected between the SWLS and the ATQ-8.

*Acceptance and Action Questionnaire-II* (AAQ-II; Bond et al., 2011; Spanish version by Ruiz et al., 2013). The AAQ-II measures general experiential avoidance through 7 items and a 7-point Likert-type scale (7 = *always*; 1 = *never true*). The items evaluate the reluctance to experience unwanted emotions and thoughts as well as the inability to be in the present moment and behave towards value-directed actions when experiencing psychological discomfort. Examples of items are: “Emotions cause problems in my life”, “I worry about not being able to control my worries and feelings”, and “It seems like most people are handling their lives better than I am”. The Spanish version by Ruiz et al. (2013) demonstrated a one-factor structure and good psychometric properties in Spanish samples with an overall alpha of .88. Alpha values in this study were .91 for both Sample 1 and Sample 2. The AAQ-II was administered because prior research has obtained strong positive correlations between ATQ scores and the AAQ-II (e.g., Ruiz & Odriozola-González, 2016).

*Cognitive Fusion Questionnaire* (CFQ; Gillanders et al., 2014; Spanish version by Ruiz et al., 2017b). The CFQ measures cognitive fusion as averaged across contexts through 7 items and a 7-point Likert-type scale (7 = *always*; 1 = *never true*), where higher scores indicate a higher degree of cognitive fusion. Examples of the items are: “I over-analyze situations to the point where it’s unhelpful to me”, “I get upset with myself for having certain thoughts”, and “I struggle with my thoughts”. The English validation of the CFQ has demonstrated to have good reliability, temporal stability, sensitivity to treatment effects, a one-factor structure, and convergent, divergent, and discriminant validity. The Spanish translation has proven to have similar psychometric properties (alpha = .92) and factor structure to the original version (Ruiz et al., 2017b). In this study, the CFQ obtained alphas of .90 and .93 for Samples 1 and 2, respectively. Medium to strong positive correlations between the CFQ and the ATQ-8 were expected.

*Generalized Pliance Questionnaire* (GPQ; Ruiz et al., 2019). The GPQ is a questionnaire consisting of 18 items, graded on a 7-point Likert-type scale (7 = *always true*; 1 = *never true*) that measures generalized pliance, defined as a pattern of rule-governed behavior in which the individual’s primary source of reinforcement is social whim. Examples of the items are: “I care a lot about what my friends think of me”, “My main goal in life is to be recognized and respected by those around me”, and “My decisions are very much influenced by other people’s opinions”. In this study, the GPQ obtained an alpha of .92 and .95 in Samples 1 and 2, respectively. Medium to strong positive correlations were expected between the GPQ and the ATQ-8.

### Procedure

For Sample 1, the instruments package was administered in the classrooms during a regular class. In Sample 2, participants answered an online survey that was advertised through social media (e.g., Facebook, institutional webpages, etc.). In both samples, participants provided written informed consent. Participants in Sample 1 responded to the following instruments: ATQ-8, DAS-R, DASS-21, SWLS, AAQ-II, CFQ, and GPQ. Participants in Sample 2 responded to the same questionnaires except for the DAS-R. Once the participants completed the study, the aims of the study were debriefed, and they were also thanked for their participation. No incentives were provided to the participants.

### Statistical and psychometric analysis

Before conducting factor analyses, the data from both samples were examined to find missing values. However, no missing data were found. Corrected item-total correlations were computed on SPSS 25© to find items that should be removed due to a low discrimination item index (i.e., values below .30). McDonald’s omega and Cronbach’s alpha were conducted to explore the ATQ-8 internal consistency with total sample (*N* = 1148) and providing percentile bootstrap confidence intervals (CI) (Viladrich, Angulo-Brunet & Doval, 2017). The MBESS package in R was used to compute these coefficients (Kelley & Lai, 2012; Kelley & Pornprasertmanit, 2016).

Because the ATQ-8 is responded on a 5-point Likert-type scale, an estimation method appropriate for ordinal data was selected to conduct the CFA. Accordingly, a robust diagonally weighted least square estimation method (Robust DWLS) was adopted using polychoric correlations. These analyses were conducted with LISREL © (version 8.71, Jöreskog & Sörbom, 1999). For the one-factor model, the chi-square test and the following goodness of fit indexes were calculated: (a) the root mean square error of approximation (RMSEA), (b) the comparative fit index (CFI), (c) the non-normed fit index (NNFI), and (d) the standardized root mean squared residual (SRMR). SRMR values below 0.05 reflect a very good fit to the data and values of 0.08 reflect a good fit to the data (Hu & Bentler, 1999; Kelloway, 1998). Kelloway (1998) suggested that values of RMSEA of 0.10 represent an acceptable or modest fit, whereas Hu and Bentler reduced the value to 0.08. Nevertheless, both guidelines suggest that a value of 0.05 reflects a very good fit to the data. Regarding the CFI and NNFI, values above .95 show a good fit to the data and above .90 indicate adequate-fitting models.

Following Jöreskog (2005) and Millsap & Yun-Tein (2004), additional CFAs were conducted to assess for metric and scalar invariances across samples, gender, groupage (younger or equal to 35 years vs. older than 35 years), and education level (primary and secondary studies vs. university studies). Metric invariance means that item factor loadings are invariant across samples, gender, groupage, and education level, whereas scalar invariance involves that item intercepts are also invariant. Consequently, a comparison was conducted among the relative fits of three increasingly restrictive models: the scalar invariance model, the metric invariance model, and the multiple-group baseline model. In so doing, we compared the relative fit of three increasingly restrictive nested models: the multiple-group baseline model (it allowed the unstandardized factor loadings to vary across groups), the metric invariance model (it placed equality of factor loadings across groups), and the scalar invariance model (it placed equality in both the factor loadings and the item intercepts across groups). For the comparison model, the indices of the CFI, NNFI, and RMSEA were compared among the nested models. Regarding the selection of a model, the more constrained model was carefully chosen (i.e., second model versus the first model, and third model versus the second model) if the following criteria proposed by Cheung & Rensvold (2002) and Chen (2007) were fulfilled: (a) the difference in RMSEA (ΔRMSEA) was lower than .01; (b) the differences in CFI (ΔCFI) and NNFI (ΔNNFI) were higher or equal to -.01.

Descriptive data were also calculated. To explore gender differences in ATQ-8 scores, an independent *t*-test was computed. Lastly, to evaluate convergent construct validity, Pearson correlations between the ATQ-8 and the other instruments were calculated.

## Results

### Descriptive data and psychometric quality of the items

Table 1 displays the Spanish translation of the items of ATQ-8 with their corrected item-total correlations for each sample and descriptive data. The eight items presented corrected item-total correlation ranging from .55 to .74 for the overall sample and good discrimination indices.

**Table 1:** Item description and corrected item-total correlations.

Item number and description	Corrected item-total correlation		
	Sample 1 Undergraduates	Sample 2 General population online	Overall sample
1. No soy Bueno [I’m no good].	.51	.58	.56
2. “Soy tan decepcionante hasta para mí mismo” [I’m so disappointed in myself].	.72	.74	.74
3. “Qué es lo que funciona mal en mí” [What’s wrong with me?].	.67	.75	.72
4. Soy un inútil, no valgo para nada [I’m worthless].	.62	.71	.70
5. Me siento tan impotente, tan desamparado [I feel so helpless].	.53	.74	.70
6. Algo tiene que cambiar [Something has to change].	.58	.71	.67
7. Mi future es un desierto [My future is bleak].	.48	.70	.66
8. No consigo terminar nada de lo que empiezo [I can’t finish anything].	.38	.59	.55

Table 2 presents the alpha and omega coefficients of the ATQ-8 for Samples 1 and 2. The alpha of the overall sample was .89 (95% CI [.88, .90]), whereas the omega was also .89 (95% CI [.88, .90]). Table 2 also shows the descriptive data of the ATQ-8. There were no statistically significant differences on the ATQ-8 scores between genders in Sample 1 (women: *M* = 16.54, *SD* = 6.92; men: *M* = 15.16, *SD* = 6.40). However, in Sample 2, women showed higher scores on the ATQ-8 than men (women: *M* = 14.39, *SD* = 5.44; men: *M* = 14.58, *SD* = 5.35).

**Table 2:** Coefficient alpha and omega, and descriptive data across samples.

	Sample 1: Undergraduates (*N*= 302)	Sample 2: General population online (*N*= 846)	Overall sample (*N*= 1,148)
Alpha [95% CI]	.83 [.80, .85]	.90 [.89, .91]	.89 [.88, .90]
Omega [95% CI]	.83 [.78, .86]	.90 [.89, .91]	.89 [.88, .90]
Mean score (*SD*)	14.46 (5.40)	16.22 (6.80)	15.76 (6.50)

### Validity evidence based on internal structure

#### Dimensionality

**Figure 1:**
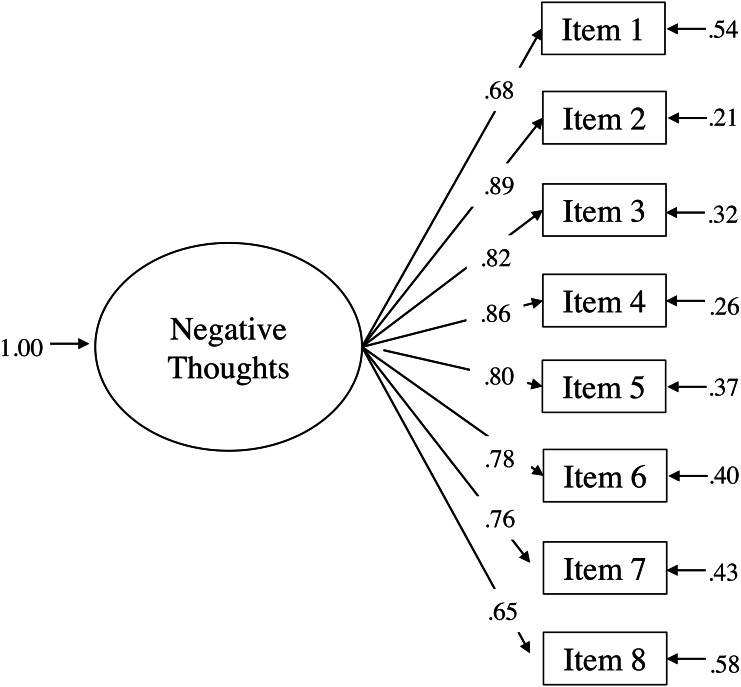
Completely standardized solution of the ATQ-8 one-factor model.

The one-factor model obtained an adequate fit according to the goodness-of-fit indexes: *χ*^2^ (20) = 251.202, *p* < .01; RMSEA = 0.10, 90% CI [0.089, 0.112], CFI = 0.98, NNFI = 0.97, and SRMR = 0.0483. Figure 1 presents the results obtained from the completely standardized solution of the one-factor model.

#### Measurement invariance

Table 3 displays the results of the analysis of the scalar and metric invariance. Changes in RMSEA, CFI, and NNFI were lower than .01 in all cases. Therefore, parameter invariance was supported at both the scalar and metric levels across samples, gender, groupage, and education level.

**Table 3:** Metric and scalar invariance across sample, gender, group age, and education level.

Model	RMSEA	ΔRMSEA	CFI	ΔCFI	NNFI	ΔNNFI
Measurement invariance across sample						
MG Baseline model	.0983		.982		.975	
Metric invariance	.1000	−.0017	.978	- .004	.974	- .001
Scalar invariance	.0984	.0016	.976	- .002	.975	.001
Measurement invariance across gender						
MG Baseline model	.101		.980		.973	
Metric invariance	.0915	.0095	.981	.001	.977	.004
Scalar invariance	.0903	.0120	.979	−.002	.978	.001
Measurement invariance across group age						
MG Baseline model	.1001		.979		.971	
Metric invariance	.1047	−.0046	.973	−.006	.968	-.003
Scalar invariance	.1089	−.0042	.966	−.007	.965	-.003
Measurement invariance across education level						
MG Baseline model	.1013		.981		.974	
Metric invariance	.1046	−.0033	.976	−.005	.972	-.002
Scalar invariance	.1033	.0013	.974	−.002	.973	.001

### Validity evidence based on relationships with other variables

Table 4 shows that the ATQ-8 presented correlations with all of the other constructs that were assessed in the expected direction: it presented positive correlations with dysfunctional schemas (DAS-R), experiential avoidance (AAQ-II), emotional symptoms (DASS-21), generalized pliance (GPQ), and cognitive fusion (CFQ); and negative correlations life satisfaction (SWLS).

**Table 4:** Pearson correlations between the ATQ-8 scores and other relevant self-report measures.

Measure	S	*N*	*r* with ATQ-8
DAS-R	1	302	.43^*^
DASS—Total	1	302	.60^*^
	2	846	.74^*^
DASS—Depression	1	302	.61^*^
	2	846	.78^*^
DASS—Anxiety	1	302	.47^*^
	2	846	.56^*^
DASS—Stress	1	302	.49^*^
	2	846	.62^*^
AAQ-II	1	302	.59^*^
	2	846	.70^*^
CFQ	1	302	.63^*^
	2	846	.65^*^
GPQ	1	302	.31^*^
	2	846	.48^*^
SWLS	2	846	−.63^*^

**Notes.**

AAQ-II Acceptance and Action Questionnaire-II ATQ-8 Automatic Thoughts Questionnaire-8 CFQ Cognitive Fusion Questionnaire DAS-R Dysfunctional Attitude Scale-Revised DASS Depression, Anxiety, and Stress Scales-21 GPQ Generalized Pliance Questionnaire SWLS Satisfaction with Life Scale

* *p* < .001.

## Discussion

While the ATQ has already been validated in Spain, to our best knowledge, no study has analyzed the factor structure and psychometric properties of the ATQ-8 with Spanish samples. This version has two main advantages over the original ATQ. Firstly, the factor structure of the ATQ-8 is more simple and stable than the one of the original ATQ. Secondly, the ATQ-8 is better suited to survey research and provides a considerably briefer assessment than the original ATQ. Accordingly, this study aimed to explore the psychometric properties of the ATQ-8 in two Spanish samples.

The analyses indicated that the Spanish version of the ATQ-8 showed good psychometric properties in Spain. Concerning internal consistency, the ATQ-8 displayed an alpha of .89, and the items had corrected item-total correlations ranging from .38 to .74. Confirmatory factor analyses showed that the one-factor model presented a good fit to the data as in the previous studies by Netemeyer et al. (2002) and Ruiz et al. (2017b). Also, the ATQ-8 showed metric and scalar measurement invariance across the type of sample (undergraduates and general online population), gender, groupage (younger or equal than 35 years vs. older than 35 years), and education level (primary and secondary studies vs. university studies). These analyses indicate that the ATQ-8 scores can be compared across these variables. Additionally, the ATQ-8 demonstrated convergent validity, given the positive correlations found with emotional symptoms, dysfunctional schemas, generalized pliance, experiential avoidance and cognitive fusion, and the negative correlations with life satisfaction.

It is worth to mention some limitations of this study. Firstly, we did not collect data from a clinical sample. This is a significant limitation because the ATQ was mainly designed to assess clinical participants. Accordingly, further studies should analyze the psychometric properties of the ATQ-8 in a clinical sample and, as in Ruiz et al. (2017b), to explore the measurement invariance across clinical and nonclinical samples. Secondly, as this study did not include a clinical sample, we were not able to analyze if the ATQ-8 can be used as a screening measure to detect unipolar depression. Thirdly, the psychometric properties of the ATQ-8 were analyzed in two convenience samples. Thus, the representativeness of the samples is uncertain. Accordingly, further studies should be conducted with other Spaniard samples to confirm the results of the current study. Fourthly, we did not explore the sensitivity to treatment. However, note that the study by Ruiz et al. (2017b) showed that the ATQ-8 was sensitive to treatment in a clinical study conducted in Colombia. Lastly, the percentage of women was significantly higher than the percentage of men in the composition of the samples. However, the finding of measurement invariance across gender reduces this limitation.

## Conclusions

The findings of the current study are consistent with previous studies by Netemeyer et al. (2002) and Ruiz et al. (2017b). Importantly, this study adds empirical evidence of the adequate fit of the one-factor structure of the ATQ-8 and its measurement invariance across gender, age, and education level. Further studies should try to replicate these findings in other Spanish-speaking countries and analyze the measurement invariance of the ATQ-8 across different cultures and countries. In conclusion, the ATQ-8 was a reliable and valid instrument in a Spanish sample. Therefore, it seems the ATQ-8 can be used in Spain as a less time-consuming measure of negative automatic thoughts than the original ATQ.

##  Supplemental Information

10.7717/peerj.9747/supp-1 Supplemental Information 1 Dataset of the study

## Additional Information and Declarations

### Data Availability

The following information was supplied regarding data availability: The raw data are available as Supplemental File.

## References

[ref-1] American Psychiatric Association (2013). Diagnostic and statistical manual of mental disorders (DSM-5®).

[ref-2] Atienza FL, Pons D, Balaguer I, García-Merita M (2000). Propiedades psicométricas de la Escala de Satisfacción con la vida en adolescentes (Psychometric properties of the satisfaction with life scale in adolescents). Psicothema.

[ref-3] Beck AT, Rush AJ, Shaw BF, Emery G (1979). Cognitive therapy of depression.

[ref-4] Bond FW, Hayes SC, Baer RA, Carpenter KM, Guenole N, Orcutt HK, Waltz T, Zettle RD (2011). Preliminary psychometric properties of the Acceptance and Action Questionnaire—II: a revised measure of psychological inflexibility and experiential avoidance. Behavior Therapy.

[ref-5] Bryant FB, Baxter WJ (1997). The structure of positive and negative automatic cognition. Cognition & Emotion.

[ref-6] Cano-García FJ, Rodríguez-Franco L (2002). Evaluación del lenguaje interno ansiógeno y depresógeno en la experiencia de dolor crónico [Assessment of anxious and depressive self-talk in chronic pain experience]. Apuntes de Psicología.

[ref-7] Chen FF (2007). Sensitivity of goodness of fit indexes to lack of measurement invariance. Structural Equation Modeling.

[ref-8] Cheung GW, Rensvold RB (2002). Evaluating goodness-of-fit indexes for testing measurement invariance. Structural Equation Modeling.

[ref-9] Chioqueta AP, Stiles TC (2004). Norwegian version of the automatic thoughts questionnaire: a reliability and validity study. Cognitive Behaviour Therapy.

[ref-10] Chioqueta AP, Stiles TC (2006). Factor structure of the dysfunctional attitude scale (Form A) and the automatic thoughts questionnaire: An exploratory study. Psychological Reports.

[ref-11] Daza P, Novy DM, Stanley M, Averill P (2002). The depression anxiety stress scale-21: Spanish translation and validation with a Hispanic sample. Journal of Psychopathology and Behavioral Assessment.

[ref-12] De Graaf LE, Roelofs J, Huibers MJ (2009). Measuring dysfunctional attitudes in the general population: the dysfunctional attitude scale (form A) revised. Cognitive Therapy and Research.

[ref-13] Deardorff PA, Hopkins LR, Finch Jr AJ (1984). Automatic thoughts questionnaire: a reliability and validity study. Psychological Reports.

[ref-14] Diener E, Emmons RA, Larsen RJ, Griffin S (1985). The satisfaction with life scale. Journal of Personality Assessment.

[ref-15] Ghassemzadeh H, Mojtabai R, Karamghadiri N, Ebrahimkhani N (2005). Psychometric properties of a Persian-language version of the Automatic Thoughts Questionnaire: ATQ-Persian. International Journal of Social Psychiatry.

[ref-16] Gillanders DT, Bolderston H, Bond FW, Dempster M, Flaxman PE, Campbell L, Remington B (2014). The development and initial validation of the Cognitive Fusion Questionnaire. Behavior Therapy.

[ref-17] Hollon SD, Kendall PC (1980). Cognitive self-statements in depression: development of an automatic thoughts questionnaire. Cognitive Therapy and Research.

[ref-18] Hollon SD, Kendall PC, Lumry A (1986). Specificity of depressotypic cognitions in clinical depression. Journal of Abnormal Psychology.

[ref-19] Hu LT, Bentler PM (1999). Cutoff criteria for fit indexes in covariance structure analysis: conventional criteria versus new alternatives. Structural Equation Modeling.

[ref-20] Jöreskog KG (2005). Structural equation modeling with ordinal variables using LISREL.

[ref-21] Jöreskog KG, Sörbom D (1999). LISREL 8.30.

[ref-22] Joseph S (1994). Subscales of the automatic thoughts questionnaire. The Journal of Genetic Psychology.

[ref-23] Kazdin AE (1990). Evaluation of the automatic thoughts questionnaire: negative cognitive processes and depression among children. Psychological Assessment.

[ref-24] Kelloway EK (1998). Using LISREL for structural equation modeling: a researcher’s guide.

[ref-25] Kelley K, Lai K (2012). http://CRAN.R-project.org/package=MBESS.

[ref-26] Kelley K, Pornprasertmanit S (2016). Confidence intervals for population reliability coefficients: evaluation of methods, recommendations, and software for composite measures. Psychological Methods.

[ref-27] Lovibond PF, Lovibond SH (1995). The structure of negative emotional states: comparison of the Depression Anxiety Stress Scales (DASS) with the beck depression and anxiety inventories. Behaviour Research and Therapy.

[ref-28] Millsap RE, Yun-Tein J (2004). Assessing factorial invariance in ordered-categorical measures. Multivariate Behavioral Research.

[ref-29] Netemeyer RG, Williamson DA, Burton S, Biswas D, Jindal S, Landreth S, Mills G, Primeaux S (2002). Psychometric properties of shortened versions of the Automatic Thoughts Questionnaire. Educational and Psychological Measurement.

[ref-30] Oei TPS, Mukhtar F (2008). Exploratory and confirmatory factor validation and psychometric properties of the automatic thoughts questionnaire for Malays (ATQ-Malay) in Malaysia. Hong Kong Journal of Psychiatry.

[ref-31] Ruiz FJ, Langer AI, Luciano C, Cangas AJ, Beltrán I (2013). Measuring experiential avoidance and psychological inflexibility: the Spanish translation of the Acceptance and Action Questionnaire. Psicothema.

[ref-32] Ruiz FJ, Martín MBG, Falcón JCS, González PO (2017a). The hierarchical factor structure of the Spanish version of Depression Anxiety and Stress Scale-21. International Journal of Psychology and Psychological Therapy.

[ref-33] Ruiz FJ, Odriozola-González P (2016). The role of psychological inflexibility in Beck’s cognitive model of depression. Anales de Psicología.

[ref-34] Ruiz FJ, Suárez-Falcón JC, Barbero-Rubio A, Flórez CL (2019). Development and initial validation of the Generalized Pliance Questionnaire. Journal of Contextual Behavioral Science.

[ref-35] Ruiz FJ, Suárez-Falcón JC, Barón-Rincón D, Barrera-Acevedo A, Martínez-Sánchez A, Pena A (2016). Factor structure and psychometric properties of the Dysfunctional Attitude Scale Revised in Colombian undergraduates. Revista Latinoamericana de Psicología.

[ref-36] Ruiz FJ, Suárez-Falcón JC, Riaño Hernández D (2017). Validity evidence of the Spanish version of the automatic thoughts questionnaire-8 in Colombia. The Spanish Journal of Psychology.

[ref-37] Ruiz FJ, Suárez-Falcón JC, Riaño Hernández D, Gillanders D (2017b). Psychometric properties of the Cognitive Fusion Questionnaire in Colombia. Revista Latinoamericana de Psicología.

[ref-38] Ruiz FJ, Suárez-Falcón JC, Odriozola-González P, Barbero-Rubio A, López-López JC, Eisenbeck N, Budziszewska LA, Gil E (2015). Factor structure and psychometric properties of the Spanish version of the Dysfunctional Attitude Scale-Revised. Psicología Conductual.

[ref-39] Sahin NH, Sahin N (1992). Reliability and validity of the Turkish version of the Automatic Thoughts Questionnaire. Journal of Clinical Psychology.

[ref-40] Viladrich C, Angulo-Brunet A, Doval E (2017). A journey around alpha and omega to estimate internal consistency reliability. Anales de Psicología.

[ref-41] Zettle RD, Webster BK, Gird SR, Wagener AL, Burdsal CA (2013). Factor structure of the Automatic Thoughts Questionnaire in a clinical sample. International Journal of Cognitive Therapy.

